# Chronic hepatitis C mortality in Brazil from 2000 to 2019: An analysis of multiple causes of death

**DOI:** 10.1016/j.clinsp.2025.100746

**Published:** 2025-09-08

**Authors:** Larissa Festa, Gerusa Maria Figueiredo, Fatima Mitiko Tengan

**Affiliations:** Faculdade de Medicina da Universidade de São Paulo (FMUSP), São Paulo, SP, Brazil

**Keywords:** Hepatitis C. Mortality. Causes of death. Brazil

## Abstract

**Introduction:**

Conventional mortality statistics that concentrate only on the underlying cause of death may offer a limited view of the complexity of this condition and not fully capture the true extent of mortality from hepatitis C.

**Objective:**

To describe and analyze deaths due to chronic hepatitis C (CHC) in Brazil, as both a underlying and multiple cause of death, from 2000 to 2019.

**Methods:**

This ecological study used data extracted from the Mortality Information System and descriptively analyzed causes of death and sociodemographic variables for the deceased.

**Results:**

During 2000–2019 there were 33,115 deaths with CHC listed on any line of the death certificate, and 25,390 (76.67 %) with CHC mentioned as the underlying cause. The authors found CHC mortality was underenumerated by 30.42 %, considering that 7725 death certificates mentioned this disease as an associated cause. Of these, liver cancer was reported as the underlying cause of 6510 deaths (84.27 %). When CHC was listed as the underlying cause, illnesses of the digestive tract were often associated with these deaths. The total number of deaths in which CHC was one of the multiple causes listed occurred predominantly in men, white people, aged 60 or over and with 12 or more years of education.

**Conclusion:**

An under-enumeration of mortality from CHC in Brazil was observed from 2000 to 2019, due to the inadequate classification of causes of death on the death certificates. Reviewing the processes of completing death certificates and coding causes is strongly recommended to obtain a more precise indicator of hepatitis C mortality.

## Highlights

• Underreporting of chronic hepatitis C deaths was identified in Brazil.
• In deaths in which Hepatitis C was the underlying cause of death, it was mentioned as an associated cause of death.
• Multiple cause analysis revealed the true extent of hepatitis C mortality.
• Study recommends reviewing the death cause recording processes in Brazil.

## Introduction

Viral hepatitis accounts for a substantial burden of disease worldwide^1^. Globally, an estimated 56.8 million people were living with Hepatitis C Virus (HCV) infection in 2020, with approximately 1.5 million new cases reported each year. This condition results in roughly 300,000 deaths annually, emphasizing the critical need for effective prevention, diagnosis, and treatment approaches^1^^,^^2^. In Brazil, the number of deaths due to hepatitis C has increased over the years, despite the stabilization trend in mortality rates over the last decade^3^.

Hepatitis C is characterized by its slow and silent progression, with chronicity occurring in 70 % to 80 % of cases^4^. Over time, the chronic stage of the disease may lead to liver damage. Initially, HCV causes liver inflammation, but its most significant consequence is the development of hepatic fibrosis, characterized by the accumulation of scar tissue in the liver^5^. Fibrosis can progress to more advanced stages, such as cirrhosis, a critical condition that increases the risk of Hepatocellular Carcinoma (HCC), a potentially fatal form of liver cancer^6^. While the primary clinical manifestations of decompensated cirrhosis are liver-related, extrahepatic manifestations attributable to HCV infection can occur regardless of liver fibrosis or cirrhosis status, ranging from mild to severe associations^7^, ^8^, ^9^. Increased all-cause mortality in patients with HCV infection is largely linked to these extrahepatic manifestations^8^. It is estimated that 74 % of chronic hepatitis C patients may experience manifestations affecting other bodily systems^7^. These manifestations include endocrine, metabolic, rheumatic, immune, renal, hematologic, cardiovascular, dermatologic, neurological, and even neoplastic disorders^7^, ^8^, ^9^, ^10^.

Studies utilizing the methodology of multiple causes of death, which analyze all causes listed on the Death Certificate (DC) ‒ underlying and associated ‒ have highlighted its promising utility in studying various chronic diseases. This approach allows for understanding the role of non-fatal diseases as contributors to death through a joint analysis of the diseases, as deaths often result from the synergistic interaction of two or more health conditions. Moreover, it enables the identification of deaths where diseases were inadequately recorded on the DC based on the patient's clinical history. Mortality statistics based on multiple causes of death improve the analysis of mortality related to the issue under study, providing a more accurate understanding and playing a significant role in assessing and planning public health actions^11^^,^^12^. Generally, traditional approaches based on single causes of death underestimate the complexity of the variety of contributing factors for the diseases under study^13^^,^^14^. In the context of hepatitis C, employing this method is justified due to the frequent positioning of this disease as an associated cause of death. According to WHO, in many cases, when individuals with severe liver disease caused by hepatitis C die from liver-related or other organ complications, the viral infection is not identified as the underlying cause of death. This implies that these deaths are attributed solely to the terminal cause, underestimating the global burden of hepatitis C^15^.

Thus, this study aims to describe and analyze deaths due to chronic hepatitis C in Brazil, as both a underlying and multiple cause of death, from 2000 to 2019.

## Methods

This study is an ecological, descriptive, and analytical investigation of deaths from chronic hepatitis C, analyzed as the underlying and multiple causes of death in Brazil from 2000 to 2019. It included deaths among Brazilian residents aged over one year, occurring between January 1, 2000, and December 31, 2019, where chronic hepatitis C was mentioned on the Death Certificate. Deaths attributed to chronic hepatitis C were defined as those containing code B18.2 of the International Statistical Classification of Diseases and Related Health Problems ‒ 10th Revision (ICD-10)^16^ in any line of Parts I and II of Field 49 of Block V (Conditions and Causes of Death) of the DC.

The Death Certificate is the official document used in Brazil to certify deaths, for both statistical and legal purposes. Block V addresses the conditions and causes of death and is divided into two parts. In Part I, composed of lines A, B, C, and D, the conditions that originated from the underlying cause of death must be declared, referred to as the consequential causes of death. The last filled line should contain the underlying cause of death, not necessarily restricted to line D. In Part II, the conditions that contributed to the death but are not part of the chain of consequential causes should be recorded, referred to as contributing causes of death. All conditions that contributed during the course of the death process (contributing causes) or were complications of the underlying cause (consequential causes), except for the underlying cause, are referred to as associated causes of death^17^.

For the analysis of multiple causes of death, only deaths with underlying causes related to hepatic and extrahepatic clinical manifestations that belong to the natural history of hepatitis C were considered. This restriction was applied to avoid overestimating deaths due to Chronic Hepatitis C (CHC) as an associated cause. Consequently, the following diseases were included, with their respective ICD-10 codes: Hepatic fibrosis and cirrhosis (K74-K74.6), Liver failure (K72-K72.9), Malignant neoplasm of the liver and intrahepatic bile ducts (C22-C22.9), Steatosis (K76.0), Hashimoto’s thyroiditis (E06.3), Malignant neoplasm of the thyroid gland (C73), Type 2 diabetes mellitus (E11, E12, E13, E14), Nutritional and metabolic disorders (E90), Arthritis (M13.9), arthralgia (M25.5), Sjögren’s syndrome (M35.0), Chronic kidney failure (N18-N18.9), nephrotic syndrome (N04-N04.9), Mixed cryoglobulinemia (D89.1), Non-Hodgkin’s B-cell lymphoma (C85-C85.9), Thrombocytopenia (D69.5-D69.6), purpura (D69-D69.9), Porphyria cutanea tarda (E80.1), Lichen planus (L43-L43.9), Atherosclerosis (I65.2, I70-I70.9), Heart failure (I50-I50.9), Ischemic stroke (I64), Chronic fatigue (G93.3), Syndromes of encephalopathy, myelitis, and encephalomyelitis (G93.4, G04.9), Depression (F32-F32.9), Gastrointestinal bleeding (K92.2).

The study area corresponds to Brazil, which is politically and administratively divided into 26 states, one Federal District, and 5570 municipalities. The country is divided into five major regions (North, Northeast, Southeast, South, and Center-West), covering an area of 8510,418 km^2^, with an estimated population of 203 million. Among the regions, the North has the largest territorial extension, comprising 3850,593 km^2^, or 45 % of the national territory. Brazil’s capital is Brasília, and São Paulo is its most populous city, located in the Southeast, with 44.4 million inhabitants^18^. The country had a per capita Gross Domestic Product (GDP) of 42,247.52 Brazilian reais in 2021 and an illiteracy rate of 5.6 % in 2022. In 2021, Brazil recorded an infant mortality rate of 11.2 deaths per thousand live births^19^.

Data on deaths of Brazilian residents were extracted from anonymized Death Certificate records available from the Mortality Information System (SIM) on the DATASUS website^20^. Population data for Brazil were obtained through population estimates from the Brazilian Institute of Geography and Statistics (IBGE), also accessible online^21^.

The variables considered in the study included: date of death, age, age group, sex, race/skin color, educational level, Federative Unit of residence, region of residence, underlying cause of death, associated causes of death (lines A, B, C, D, and Part II), and multiple causes of death.

Completeness analysis was performed for the following study variables: date of death, age, sex, race/skin color, educational level, underlying cause of death, and associated causes of death (lines A, B, C, D, and Part II), using absolute and relative frequencies. Subsequently, a descriptive analysis of diseases associated with CHC as the underlying cause of death and of diseases as primary causes when CHC was an associated cause of death was conducted according to ICD-10 chapters, based on absolute and relative frequencies.

The sociodemographic characteristics of deaths from chronic hepatitis C, as the underlying and multiple causes of death, were analyzed using absolute and relative frequencies of variables including sex, age group, race/skin color, and educational level.

Secondary databases of public access were utilized, without information enabling the identification of individuals, in accordance with ethical principles established by Resolution No. 466, dated December 12, 2012, of the National Health Council (CNS) regarding research involving human subjects.

This study was carried out with the support of CAPES, a Brazilian Government agency dedicated to the training of human resources.

## Results

**Fig. 1:**
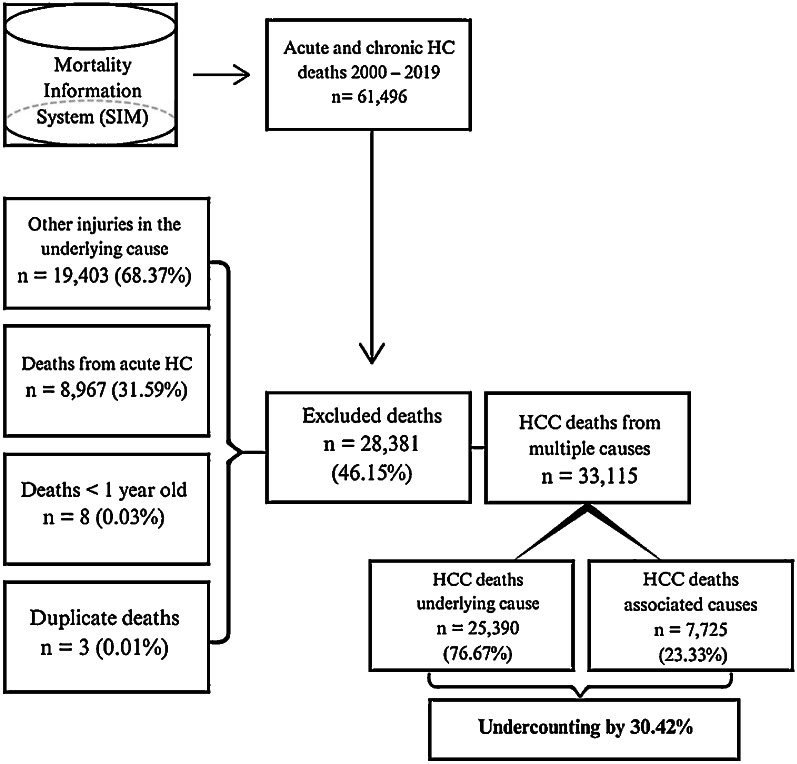
Flowchart of the steps for database preparation and study population selection.

Between 2000 and 2019, data extracted from the Mortality Information System (SIM) indicated 61,496 deaths from hepatitis C, in either acute or chronic forms, recorded on Death Certificates as the underlying or associated causes of death (consequential and contributing causes). Applying the eligibility criteria defined in this study led to the exclusion of 28,381 (46.15 %) deaths. Of these, 19,403 (68.37 %) were deaths that did not meet the criteria for CHC to be considered as an associated cause of death; 8967 (31.59 %) were deaths due to the acute form of the disease; 8 (0.03 %) were deaths of individuals under one year of age; and 3 (0.01 %) were duplicate records. The final database comprised 33,115 deaths from CHC in any line of the DC, with 25,390 (76.67 %) recorded as the underlying cause of death and 7725 (23.33 %) as an associated cause of death. Calculating the representativeness of CHC as an associated cause of death relative to its designation as the underlying cause of death (7725/25,390×100) revealed that mortality from CHC as the underlying cause was underestimated by 30.42 % (Fig. 1).

Table 1 presents the sociodemographic profile of deaths from chronic hepatitis C in Brazil from 2000 to 2019, categorized by whether the disease was mentioned as the underlying or an associated cause of death. From a total of 33,115 deaths, 20,590 (62.2 %) were male, while 12,522 (37.8 %) were female. The proportion of males was higher among deaths where CHC was mentioned as an associated cause, with 5413 deaths (70.1 %), compared to its mention as a underlying cause, with 15,177 deaths (59.8 %). Notably, three deaths were recorded with the sex classified as “unknown”. Regarding age, deaths from CHC as a multiple cause of death were predominantly among individuals aged 60-years or older, accounting for 17,905 (54.1 %) deaths. A similar pattern was observed when CHC was analyzed as the underlying cause of death 13,425 (52.9 %) and as an associated cause 4480 (58.0 %), with the latter category showing the highest proportion for this age group. Concerning race/skin color, deaths from CHC as a multiple cause of death were predominantly among white individuals 22,811 (68.9 %), followed by individuals of mixed race 6721 (20.3 %). These proportions were similar when analyzed according to CHC as a underlying or associated cause of death. In terms of educational level, 8361 (25.3 %) individuals who died from CHC as a multiple cause of death had 12 or more years of schooling. This proportion was higher among deaths from CHC recorded as an associated cause of death (30.3 %).

**Table 1:** 

Sociodemographic characteristics	Total deaths (MC)		Hepatitis C underlying cause		Hepatitis C associated cause	
	(n=33,115)		(n=25,390)		(n=7,725)	
	n	%	n	%	n	%
**Sex**						
Female	12,522	37.8	10,210	40.2	2,312	29.9
Male	20,590	62.2	15,177	59.8	5,413	70.1
Ignored/blank	3	0.0	3	0.0	0	0.0
**Age group**						
0 - 14	7	0.0	6	0.0	1	0.0
15 - 29	174	0.5	157	0.6	17	0.2
30 - 39	839	2.5	734	2.9	105	1.4
40 - 49	4,132	12.5	3,430	13.5	702	9.1
50 - 59	10,053	30.4	7,634	30.1	2,419	31.3
≥ 60	17,905	54.1	13,425	52.9	4,480	58.0
Ignored/blank	5	0.0	4	0.0	1	0.0
**Race/color**						
White	22,811	68.9	14,497	68.9	5,314	68.8
Black	2,012	6.1	1,473	5.8	539	7.0
Brown	6,721	20.3	5,221	20.6	1,500	19.4
Yellow	252	0.8	163	0.6	89	1.2
Indigenous	22	0.1	19	0.1	3	0.0
Ignored/blank	1,297	3.9	1,017	4.0	280	3.6
**Schooling**						
None	797	2.4	636	2.5	161	2.1
1 to 3	2.338	7.1	1.882	7.4	456	5.9
4 to 7	6.635	20.0	5.417	20.3	1.488	19.3
8 to 11	6.433	19.4	4.909	19.3	1.524	19.7
12 or more	8.361	25.3	6.023	23.7	2.338	30.3
Ignored/blank	8.551	25.8	6.793	26.8	1.758	22.8

Source: SIM – Brasil, 2021.

The variables used in this study were described according to completeness, considering the mention of hepatitis C as a multiple cause of death. None of the variables showed completeness below 50 %. The variables “Date of death” and “Underlying cause” had no missing or blank data. However, the variables “Part II” and “Line D” had the lowest completeness rates, with 54 % and 53.8 %, respectively. Among sociodemographic variables, “Educational level” had the lowest completeness rate, with 25.8 % of fields blank or marked as “unknown” (Table 2).

**Table 2:** Distribution of Death Certificates mentioning chronic hepatitis C as multiple causes of death, by completeness per variable, Brazil, 2000–2019.

Variable	Ignored/Blank		Completeness	
	n	%	n	%
Date of death	0	0	33,115	100.0
Underlying cause	0	0	33,115	100.0
Sex	3	0	33,112	99.9
Age	5	0	33,110	99.9
Race/Color	1297	3.9	31,818	96.1
Line A	1903	5.7	31,212	94.3
Line B	2114	6.4	31,001	93.6
Line C	5331	16.1	27,784	83.9
Education	8551	25.8	24,564	74.2
Part II	15,239	46.0	17,876	54.0
Line D	15,301	46.2	17,814	53.8

Source: SIM – Brazil, 2021.

Among the 7725 deaths where CHC was recorded as an associated cause, more than half had neoplasms (ICD-10 Chapter II) as the underlying cause (6671 86.36 %), followed by diseases of the circulatory system (Chapter IX; 374; 4.84 %) and digestive system diseases (Chapter XI; 292; 3.78 %) (Table 3).

**Table 3:** Distribution of deaths from chronic hepatitis C as an associated cause of death, by underlying causes recorded according to ICD-10 chapters, Brazil, 2000–2019.

Chapter and description ICD-10	n	%
Chapter II (Neoplasms)	6671	86.36
Chapter III (Diseases of the Blood and Hematopoietic Organs and Some Immune Disorders)	40	0.52
Chapter IV (Endocrine, Nutritional and Metabolic Diseases)	101	1.31
Chapter V (Mental and Behavioral Disorders)	1	0.01
Chapter VI (Diseases of the Nervous System)	14	0.18
Chapter IX (Diseases of the Circulatory System)	374	4.84
Chapter XI (Diseases of the Digestive System)	292	3.78
Chapter XIII (Diseases of the Musculoskeletal System and Connective Tissue)	1	0.01
Chapter XIV (Diseases of the Genitourinary System)	231	2.99
**Total**	**7725**	**100.00**

Source: SIM – Brazil, 2021.

The most frequent diseases within these three chapters are detailed in Table 4. Among neoplasms, malignant neoplasms of the liver and intrahepatic bile ducts (C22 to C22.9) accounted for 6510 (84.27 %) deaths. Within circulatory system diseases, heart failure (I50 to I50.9) was the most common, with 144 (1.86 %) deaths, followed by esophageal varices (I85 to I85.9); (120; 1.55 %), and ischemic stroke (I64), (104; 1.35 %). In digestive system diseases, spontaneous bacterial peritonitis (K65 to K65.9) was the most frequent underlying cause, 218 (2.82 %), followed by hepatic fibrosis and cirrhosis (K74 to K74.6); 51 (0.66 %) deaths.

**Table 4:** Distribution of deaths from chronic hepatitis C as an associated cause of death, by underlying causes within the three most frequent ICD-10 chapters, Brazil, 2000–2019.

ICD-10 chapter, description and code	n	%
**Chapter II – Neoplasms**		
Malignant neoplasm of liver and intrahepatic bile ducts ‒ C22 to C22.9	6.510	84,27
Non-Hodgkin B-cell lymphomas ‒ C85 to C85.9	157	2,03
Malignant neoplasm of thyroid gland ‒ C73	4	0,05
**Chapter IX – Diseases of the Circulatory System**		
Heart failure ‒ I50 to I50.9	144	1,86
Esophageal varices ‒ I85 to I85.9	120	1,55
Ischemic stroke ‒ I64	104	1,35
Gastric varices ‒ I86.4	5	0,06
Atherosclerosis ‒ I65.2, I70 to I70.9	1	0,01
**Chapter XI – Diseases of the Digestive System**		
Spontaneous bacterial peritonitis K65 to K65.9	218	2,82
Hepatic fibrosis and cirrhosis K74 to K74.6	51	0,66
Hepatic failure ‒ K72 to K72.9	16	0,21
Steatosis ‒ K76.0	4	0,05
Portal hypertension ‒ K76.6	1	0,01
Hepatorenal syndrome ‒ K76.7	1	0,01
Gastrointestinal bleeding ‒ K92.2	1	0,01
**Other Chapters**^a^	388	5,02
**Total**	**7725**	**100,00**

a Various ICD-10 codes. Source: SIM – Brazil, 2021.

Of the 25,390 deaths in which CHC was recorded as the underlying cause of death, there were 81,206 mentions of associated causes, both consequential and contributing. The diseases most commonly recorded as associated causes of death corresponded to the digestive system, with 36,995 (45.56%) mentions, followed by symptoms, signs, and abnormal clinical and laboratory findings not elsewhere classified, with 9552 (11.76%) mentions, and infectious and parasitic diseases, with 7151 (8.81%) mentions (Table 5). Given that Chapter XI of ICD-10 (Diseases of the Digestive System) showed the highest frequency of mentions of associated causes when CHC was the underlying cause, Table 6 describes the most frequent diseases within this chapter. Of the total 36,995 mentions of diseases affecting the digestive system, it was observed that hepatic fibrosis and cirrhosis (K74 to K74.6) had the highest number of mentions as associated causes, with 16,370 (44.25%) mentions.

**Table 5:** Distribution of deaths from chronic hepatitis C as the underlying cause of death, according to mentions of associated causes of death by ICD-10 chapters, Brazil, 2000–2019.

ICD-10 chapter, description and code	n	%
Chapter I (Some Infectious and Parasitic Diseases) ‒ A00 to B99	7151	8.81
Chapter II (Neoplasms) ‒ C00 to D48	1671	2.06
Chapter III (Diseases of the Blood and Blood-Forming Organs and some Immune Disorders) ‒ D50 to D89	1192	1.47
Chapter IV (Endocrine, Nutritional and Metabolic Diseases) ‒ E00 to E90	3096	3.81
Chapter V (Mental and Behavioural Disorders) ‒ F00 to F99	1775	2.19
Chapter VI (Diseases of the Nervous System) ‒ G00 to G99	380	0.47
Chapter VII (Diseases of the Eye and Adnexa) ‒ H00 to H59	4	0.00
Chapter VIII (Diseases of the Ear and Mastoid Process) ‒ H60 to H95	1	0.00
Chapter IX (Diseases of the Nervous System) Circulatory System) ‒ I00 to I99	5966	7.35
Chapter X (Diseases of the Respiratory System) ‒ J00 to J99	6336	7.80
Chapter XI (Diseases of the Digestive System) ‒ K00 to K93	36,995	45.56
Chapter XII (Diseases of the Skin and Subcutaneous Tissue) ‒ L00 to L99	162	0.20
Chapter XIII (Diseases of the Musculoskeletal System and Connective Tissue) ‒ M00 to M99	112	0.14
Chapter XIV (Diseases of the Genitourinary System) ‒ N00 to N99	4535	5.58
Chapter XVI (Some Conditions Originating in the Perinatal Period) ‒ P00 to P96	2	0.00
Chapter XVII (Congenital Malformations, Deformities and Chromosomal Anomalies) ‒ Q00 to Q99	27	0.03
Chapter XVIII (Symptoms, Signs and Abnormal Findings, Not Classified under Other Part) ‒ R00 to R99	9552	11.76
Chapter XIX (Injuries, Poisoning and Certain Other Consequences of External Causes) ‒ S00 to T98	781	0.96
Chapter XX (External Causes of Morbidity and Mortality) ‒ V01 to Y98	1439	1.77
Chapter XXI (Factors Influencing Health Status and Contact with Health Services) - Z00 to Z99	29	0.04
**Total**	**81,206**^a^	**100.00**

a Total mentions of diseases as associated causes excluding chronic hepatitis C. Source: SIM – Brazil, 2021.

**Table 6:** Distribution of deaths from chronic hepatitis C as the underlying cause of death, according to the most frequent associated causes of death within Chapter XI of the ICD-10, Brazil, 2000–2019.

Chapter XI - Diseases of the Digestive System	n	%
Fibrosis and cirrhosis of the liver (K74 to K74.6)	16,370	44.25
Hepatic failure, not elsewhere classified (K72 to K72.9)	8594	23.23
Other diseases of the digestive system (K92 to K92.9)	5169	13.97
Other diseases of the liver (K76 to K76.9)	4002	10.82
Peritonitis (K65 to K65.9)	1860	5.03
Other diseases of the chapter^a^	1000	2.70
**Total**	**36,995**	**100.00**

a Various ICD-10 codes. Source: SIM – Brazil, 2021.

## Discussion

This study’s analysis of Chronic Hepatitis C (CHC) deaths, considering the multiple causes of death listed on Death Certificates (DCs), provided a comprehensive understanding of mortality trends and distribution associated with this condition in Brazil. Including 7725 death records where CHC was mentioned as an associated cause of death revealed a 30.42 % underreporting of mortality linked to this condition compared to deaths where CHC was exclusively listed as the underlying cause.

Employing the multiple-cause-of-death analysis for hepatitis C proved successful in various national and international contexts, contributing to a deeper understanding of mortality associated with this disease^22^, ^23^, ^24^, ^25^. A study conducted in Massachusetts, USA, from 2002 to 2011 examined the DCs of residents mentioning hepatitis C and/or HIV/AIDS. In this analysis, all listed causes of death were considered, revealing that hepatitis C was often reported as an associated cause of death, contrasting with HIV/AIDS, which was predominantly identified as the underlying cause^24^.

The frequent attribution of hepatitis C as an associated cause of death is occasionally debated. Literature addressing hepatitis C-associated mortality emphasizes the recurrent issue of underrecognizing this viral infection as the underlying cause of death^22^, ^23^, ^24^. This could be attributed to the complexity of determining the cause of death related to this condition, as the chronic stage may involve various manifestations in the body. While the liver is the main organ affected, numerous extrahepatic conditions can arise due to HCV infection^7^, ^8^, ^9^, ^10^. Consequently, infected individuals may experience the infection’s effects even without severe liver disease^23^.

However, according to the World Health Organization (WHO), this underrecognition persists even in cases involving individuals with HCV infection who die in the terminal stages of hepatic complications such as decompensated cirrhosis and hepatocellular carcinoma^15^. These findings highlight the importance of acknowledging that traditional mortality statistics focusing solely on the underlying cause of death may underestimate the burden of hepatitis C mortality^24^.

The issue is further compounded by inaccuracies in the documentation of causes of death on DCs due to errors such as improper completion, misplacement of information, or coding inaccuracies^26^. These documentation errors are observed globally and can significantly distort the understanding of the actual causes of death, compromising the quality of vital statistics^27^. For instance, a comparative study conducted in the United States between clinical and autopsy-based DCs found that 41 % of certificates had incorrect cause-of-death entries, with the underlying issue being the inappropriate arrangement of information in Part I^28^. In Brazil, a retrospective cross-sectional study in Belém analyzed 800 DCs and found that 71.5 % contained inaccuracies in completing the underlying cause of death field. The most common error was using “garbage codes”, which refer to causes of death that provide non-specific and imprecise information^29^^,^^30^.

In this context, international research identified specific factors related to the professionals responsible for completing DCs, such as advanced age, educational background, and medical specialty, as contributors to inaccuracies in determining the underlying cause of death^27^^,^^31^. In Brazil, an exploratory study in Belo Horizonte revealed that 68 % of 44 % surveyed physicians reported difficulties completing DCs accurately, with 11 % citing unclear instructions as the underlying reason^32^.

Regarding the completeness of the variables used in this study, data from the SIM ‒ a well-established health information system in Brazil ‒ ensured a high degree of variable completeness. Although “Part II” and “Line D” had the highest percentage of blank or unknown fields, overall, the database variables demonstrated excellent completeness. It is important to note, however, that completing these two variables is not mandatory in all cases. The sequence of events leading to death may be fully described before Line D, and fatalities may not involve contributing conditions requiring documentation in Part II.

This study focused exclusively on deaths related to the chronic phase of hepatitis C, enabling a more accurate assessment of mortality attributable to this disease in Brazil. The acute HCV infection phase tends to be self-limiting, resolving spontaneously before progressing to chronicity, making deaths due to acute hepatitis C extremely rare^33^. Thus, a substantial proportion of deaths corresponding to the acute form of hepatitis C, mentioned in the causes of death, whether as the underlying or associated cause, was excluded during database construction. The Ministry of Health’s Viral Hepatitis Epidemiological Bulletin considers both acute and chronic forms when preparing mortality statistics for this disease^3^. Mentioning acute hepatitis C as the underlying cause of death on DCs could result from possible errors in completion and/or coding. Nevertheless, it is critically important to recognize the need for specific studies to clarify the underlying circumstances of this issue.

Currently, WHO employs a hepatitis C-related mortality indicator that incorporates deaths from decompensated cirrhosis and hepatocellular carcinoma in its calculation, as these are the main causes of death stemming from chronic HCV infection^34^. This study confirmed a significant relationship between liver cancer and hepatitis C. In cases where CHC was recorded as an associated cause of death, liver cancer was the most frequently recorded underlying cause of death (84.27 %). Similarly, in São Paulo, concordant results were found, with liver cancer identified as the underlying cause in 59.9 % of deaths where hepatitis C was mentioned as an associated cause^22^. From another perspective, a study analyzing CHC deaths in São Paulo State from 2009 to 2017 found that of 5870 deaths where liver cancer was identified as the underlyingcause, 22 % were associated with hepatitis C^35^.

However, the high proportion of Hepatocellular Carcinoma (HCC) deaths due to HCV infection raises concerns that deaths from hepatitis C may be significantly underestimated as a underlying cause in cases involving liver cancer. According to Sena (2023)^25^, this underestimation may stem from two primary factors: a lack of recognition of the causal relationship between these two diseases by physicians when completing the causes of death on DCs or the coding process for causes of death performed by the Brazilian Mortality Information System (SIM).

As highlighted by the author, SIM guidelines do not consider hepatitis C as a cause capable of leading to liver cancer. Consequently, even when hepatitis C is appropriately listed in the causal sequence as the underlying cause, it is often disregarded by the system in favor of the cancer diagnosis^25^. This scenario raises significant concerns about the accuracy of documenting deaths related to hepatitis C and underscores the need to improve coding rules to ensure proper recording of these causes of death.

Following liver cancer, the most frequent underlying causes of death when hepatitis C was mentioned as an associated cause were spontaneous bacterial peritonitis (2.82 %), non-Hodgkin B-cell lymphoma (2.03 %), and heart failure (1.86 %). Deaths attributed to spontaneous bacterial peritonitis may be explained by this complication’s occurrence in patients with liver disease due to cirrhosis. A systematic review and meta-analysis of ten studies involving 1713 cases of cirrhosis caused by viral infection across eight countries found a prevalence of 4 % for spontaneous bacterial peritonitis among patients with HCV-related cirrhosis^36^.

On the other hand, HCV infection has been associated with an increased risk of non-Hodgkin B-cell lymphoma, which is considered an extrahepatic manifestation of chronic HCV infection. A retrospective cohort study in Taiwan using a national database observed that the incidence of non-Hodgkin lymphoma among patients with hepatitis C was significantly higher than among the general population (RR = 2.36; 95 % CI 1.73–3.22)^37^.

These findings suggest the possibility that deaths from spontaneous bacterial peritonitis and B-cell non-Hodgkin lymphoma, even if occurring in small proportions, may also underestimate hepatitis C mortality due to the potential lack of recognition of these diseases as consequences of HCV infection.

The presence of heart failure as a underlying cause of death in cases where CHC was an associated cause, even in low proportions, represents a public health concern. This outcome is classified as a “garbage code^30^”. Although evidence indicates that cardiovascular diseases, such as carotid atherosclerosis, ischemic stroke, and heart failure, have been associated with HCV infection and are considered potential extrahepatic manifestations, their etiology is multifactorial. Therefore, it is not appropriate to attribute them solely to hepatitis C or document them as the underlyingcause of death based only on this association.

The incorrect documentation of underlying causes in deaths identified in this study, when they did not play a principal role in triggering the series of subsequent events, distorts mortality statistics and impairs the accurate understanding of hepatitis C mortality in Brazil. This compromises adequate health condition assessments and undermines the formulation of effective public health policies.

Fibrosis and cirrhosis, major complications arising from HCV infection, were recorded as the underlying cause of death in only 0.66 % of cases in this study. In contrast, when CHC was documented as the underlying cause of death, the most frequent associated causes belonged to Chapter XI of the ICD-10 (Diseases of the Digestive System), accounting for 45.56 % of mentions. This result aligns with existing literature, as HCV primarily targets the liver, an organ of the digestive system, and its main clinical manifestations are liver-related^4^. These manifestations include conditions such as hepatic steatosis, fibrosis, cirrhosis, liver failure, and HCC^5^. Among these diseases, fibrosis and cirrhosis were the most frequently associated with CHC (44.25 %). This proportion reflects the correct completion of the chain of events leading to death on DCs, as approximately 10 %–20 % of individuals chronically infected with HCV develop cirrhosis as a consequence of the infection (Table 5, Table 6)^38^.

It is worth noting that the inclusion criteria for this study excluded hepatic conditions associated with alcohol consumption, such as alcoholic cirrhosis. This decision was made to avoid distortions in the results, as alcoholic cirrhosis is a consequence of excessive alcohol use. While literature suggests that individuals with HCV infection and alcohol abuse exhibit higher mortality rates than those abusing alcohol without HCV infection, this aggravation is not part of the natural history of hepatitis C. Instead, it represents a contributing factor that exacerbates liver damage progression^39^^,^^40^.

Among the 33,115 CHC deaths recorded in Brazil from 2000 to 2019, the sociodemographic profile was consistent, regardless of whether the disease was listed as the underlying or associated cause of death. The majority of deaths occurred among males, individuals of white race/skin color, people aged 60-years or older, and those with 12 or more years of education. Similar profiles have been observed in other parts of the country, such as the Federal District and the city of São Paulo^22^^,^^25^.

In the Federal District, an analysis of deaths from 2006 to 2020 that mentioned hepatitis C in any line of the DC found a predominance of males and white individuals. Additionally, the most affected age group was 50–69 years, and most patients had incomplete higher education^25^. In São Paulo, from 2002 to 2016, of the 3194 deaths where hepatitis C was listed as the underlying cause, 55.10 % were men, with an even higher predominance of white individuals (76.02 %). However, regarding education, most had fewer than eight years of schooling (48.90 %), and only 13.02 % had completed 12 or more years of education^22^.

The death profile concerning sex and age found in this study is consistent with international results^23^^,^^41^^,^^42^. In Europe, a national retrospective population-based analysis conducted in Poland identified that most CHC deaths registered from 2009 to 2021 occurred among males aged 45–64^41^. Similarly, in the United States in 2010, a study analyzing 18,473 deaths reported with viral hepatitis found that hepatitis C was the underlying cause in approximately 90 % of cases, primarily affecting individuals aged 45–64^42^. Furthermore, in an American cohort tracking 21,378 individuals with chronic HCV infection between 2011 and 2017, the average age at death was 60^23^.

The high occurrence of CHC-related deaths among older individuals may be associated with the “Baby Boomer” generation, comprising those born between 1945 and 1965. This period was marked by a sharp increase in birth rates after World War II, particularly in participating countries. HCV has been widely identified in this generation due to their higher susceptibility to virus transmission during their youth, particularly in the 1970s and 1980s. This increased susceptibility is attributed to higher engagement in risky practices, such as intravenous drug use and high-risk sexual activities with multiple partners^43^. However, further studies are needed to investigate this phenomenon’s influence on hepatitis C in the Brazilian context.

On the other hand, this study found rare occurrences of CHC-related deaths in individuals under 14. This finding can be attributed to the disease’s nature, which progresses slowly in the body. Severe complications leading to death generally develop after decades of chronic infection^38^. In São Paulo, this characteristic was mirrored, as no deaths were recorded among those under 15^22^.

The predominance of deaths among white individuals highlights a significant disparity relative to Brazil’s demographics, where 56 % of the population identifies as Black or mixed race^44^. Moreover, the high proportion of deceased individuals with 12 or more years of education is notable. The literature indicates that education level and income are associated with racial and ethnic disparities among HCV-infected individuals^23^^,^^43^. Revisiting Spradling et al.'s (2021) study[23] in the United States, it was observed that among deceased individuals with HCV infection, those less likely to have hepatitis C documented on their DCs were African American, low-income individuals, and those without private health insurance. These characteristics are frequently associated with disparities faced by Black and mixed-race individuals in accessing both primary and specialized healthcare. This gap can lead to scenarios where hepatitis C diagnoses are neglected at the time of death^23^. In Brazil, despite the principles of universality and comprehensiveness promoted by the SUS (Unified Health System), aiming to mitigate racial inequalities in healthcare access, these disparities persist^45^.

Understanding the socioeconomic aspects of hepatitis C-related mortality is crucial for guiding efforts to reduce missed opportunities for diagnosis and treatment. Nevertheless, there is global evidence of changing epidemiology in HCV infections, particularly in developed countries. These changes are largely attributed to increased intravenous drug use, potentially contributing to rising cases and deaths among young adults, regardless of race, ethnicity, or gender^43^.

## Conclusions

This study identified a 30.42 % underreporting of Chronic Hepatitis C (CHC) mortality in Brazil during the period from 2000 to 2019 when comparing deaths where CHC was mentioned only as the underlying cause to those where it was included as an associated cause of death. By considering multiple causes of death, the analysis provided a more comprehensive and accurate perspective of mortality related to CHC compared to traditional methods focusing solely on underlying causes.

The study revealed that among the various hepatic and extrahepatic manifestations that can develop during the clinical course of hepatitis C, malignant neoplasms of the liver were most frequently recorded as the underlying cause of death where CHC was an associated cause. These results contribute to a better understanding of the mortality reality associated with the chronic form of hepatitis C in Brazil. By incorporating deaths where CHC was erroneously positioned as an associated cause, the analyses provided a more accurate and comprehensive view of mortality from this disease.

This highlights the need to review the processes for completing and coding causes of death on Death Certificates to improve the hepatitis C mortality indicator in the country. Furthermore, the findings emphasize the importance of future research exploring multiple causes of death, not only in the context of hepatitis C but also for other diseases of public health significance.

## Justifications

The data used in this study are secondary and sourced from publicly available, anonymized databases. Therefore, informed consent was not required, in accordance with ethical guidelines for research using publicly available data, and submission to a Research Ethics Committee is not required.

The use of >40 references is justified by the complexity of the topic, which requires a solid foundation in epidemiological, methodological, and regulatory aspects. The citations are essential to contextualize the hepatitis C scenario, support the multiple cause-of-death approach, compare findings with previous studies, and substantiate recommendations based on national and international guidelines. All references were carefully selected for their direct relevance to the article’s arguments and conclusions.

## Authors' contributions

Festa L: Conceptualization; methodology; software; validation; formal analysis; investigation; data curation; writing-original draft; writing-review & editing, visualization, project administration, funding acquisition.

Tengan FM: Conceptualization; methodology; writing-review; editing; supervision.

Figueiredo GM: Conceptualization; methodology; validation; writing-review & editing; supervision; project administration; funding acquisition.

## Declaration of competing interest

The authors declare no conflicts of interest.
